# Identification of Differences in Body Composition Measures Using 3D-Derived Artificial Intelligence from Multiple CT Scans across the L3 Vertebra Compared to a Single Mid-Point L3 CT Scan

**DOI:** 10.1155/2023/1047314

**Published:** 2023-10-17

**Authors:** Ke Cao, Josephine Yeung, Yasser Arafat, Matthew Y. K. Wei, Justin M. C. Yeung, Paul N. Baird

**Affiliations:** ^1^Department of Surgery, Western Precinct, University of Melbourne, Melbourne, Australia; ^2^Department of Colorectal Surgery, Western Health, University of Melbourne, Melbourne, Australia; ^3^Department of Surgery, University of Melbourne, Melbourne, Australia

## Abstract

**Purpose:**

Body composition analysis in colorectal cancer (CRC) typically utilises a single 2D-abdominal axial CT slice taken at the mid-L3 level. The use of artificial intelligence (AI) allows for analysis of the entire L3 vertebra (non-mid-L3 and mid-L3). The goal of this study was to determine if the use of an AI approach offered any additional information on capturing body composition measures.

**Methods:**

A total of 2203 axial CT slices of the entire L3 level (4–46 slices were available per patient) were retrospectively collected from 203 CRC patients treated at Western Health, Melbourne (97 males; 47.8%). A pretrained artificial intelligence (AI) model was used to segment muscle, visceral adipose tissue (VAT), and subcutaneous adipose tissue (SAT) on these slices. The difference in body composition measures between mid-L3 and non-mid-L3 scans was compared for each patient, and for males and females separately.

**Results:**

Body composition measures derived from non-mid-L3 scans exhibited a median range of 0.85% to 6.28% (average percent difference) when compared to the use of a single mid-L3 scan. Significant variation in the VAT surface area (*p* = 0.02) was observed in females compared to males, whereas male patients exhibited a greater variation in SAT surface area (*p* < 0.001) and radiodensity (*p* = 0.007).

**Conclusion:**

Significant differences in various body composition measures were observed when comparing non-mid-L3 slices to only the mid-L3 slice. Researchers should be aware that considering only the use of a single midpoint L3 CT scan slice will impact the estimate of body composition measurements.

## 1. Introduction

The measurement of body composition relies on the assessment of quantity and distribution of body fat and lean muscle mass [1] and varies between sexes [2]. In colorectal cancer (CRC) patients, body composition has been associated with survival-related clinical outcomes [3–8]. The most common technique for evaluating body composition has arisen through the use of computed tomography (CT) [9]. Grading of CT images through the use of a semiautomated analysis using a manual interpretation of body composition is possible, but this approach has limitations due to its labour-intensive nature and a high degree of specialisation. A single abdominal axial CT image taken at the L3 level (typically at the midpoint of L3, referred to as mid-L3 from hereon) is typically used to examine body composition in individuals with CRC [10–12]. However, there is limited justification as to why the mid-L3 is used as the gold standard [13, 14] and limited data exist to compare whether body composition measures utilising other CT slices from L3 or the entire L3 vertebral level (non-mid-L3) result in different estimates.

Deep learning is one of the primary techniques used in artificial intelligence (AI), and its use has been growing in popularity as a viable approach for automating the process of body composition segmentation [15]. In prior studies, AI models designed to replicate the process of semiautomated analysis have been trained and validated using a single mid-L3 slice [16–21]. These models have yielded promising results [16–21]. Our previously trained AI model has also shown promising segmentation (98% dice similarity) of CT body composition in CRC patients (submitted for publication). The use of AI technologies may therefore make it possible for the rapid acquisition of other L3 slices to assess body composition measures compared to those from a single mid-L3 slice.

In the present study, we aimed to employ our in-house AI model for automated segmentation and quantification of body composition from all available CT scans from a patient's complete L3 level. This would allow determination as to the level of variation across the L3 region in terms of estimating body composition measurements and highlight any potential impact on future clinical studies.

## 2. Methods

This study was approved by the Western Health Office for Research (Project QA2020.24_63907). The protocol followed the tenets of the Declaration of Helsinki and all privacy requirements were met.

The AI model was developed and validated using Python 3.7.11, Spyder 5.15 (Anaconda distribution) with Keras (https://keras.io/) and Tensorflow (https://www.tensorflow.org/) using NVIDIA RTX Graphics Processing Unit. RStudio (version 2022.2.2.485) was used to perform other statistical analysis.

### 2.1. Study Population and CT Scans

Using sagittal imaging, the anatomical level of L3 was identified by a trained human grader (author JoY) using the medical image viewer Synapse 5 (FUJIFILM). All available axial scans (*n* = 2203 axial scans) at the L3 level for each patient were collected. For each patient, one CT slice being most representative of the L3 was defined as the mid-L3 slice, which in line with the Alberta Protocol (https://tomovision.com/Sarcopenia_Help/index.htm) was manually selected by a trained human grader (author JoY).

Each collected CT scan was represented as a digital imaging and communications in medicine (DICOM) image with a resolution of 512 by 512 pixels. The CT scan parameters included slice thickness (1 mm–8 mm) and dose value (100–140 kVp) that differed depending on the clinical indication. Each CT unit/pixel was transformed to the Hounsfield unit (HU) scale; a quantitative measure of radiodensity for analysing CT scans [22] using the formula: pixel value × slope + intercept (https://www.idlcoyote.com/fileio_tips/hounsfield.html). The pixel value, intercept, and slope were retrieved from each DICOM file.

Patients' inclusion criteria included being (a) diagnosed with colon cancer at Western Health between 2012 and 2021. Patients were identified from the Australian Comprehensive Cancer Outcomes and Research Database (ACCORD), a prospectively maintained registry of patients diagnosed with CRC in Victoria, Australia; (b) availability of L3 axial CT scans.

Patients were excluded from the study if any of the following were present in their L3 scan: (a) low CT scan quality that was difficult to manually read; (b) evidence of an excess quantity of SAT extending outside the CT image; (c) signs of muscle cut off; and (d) presenting with major artefacts.

Age at the time of diagnosis and sex were both obtained from the ACCORD database for each patient.

### 2.2. Body Composition Measures

This study examined skeletal muscle (SM), visceral adipose tissue (VAT), and subcutaneous adipose tissue (SAT) as components of body composition measures on the mid-L3 slice and other L3 slices for each patient. The following body composition measures were analysed in this study:

1. SM surface area (cm^2^)
2. VAT surface area (cm^2^)
3. SAT surface area (cm^2^)
4. SM radiodensity (HU)
5. VAT radiodensity (HU)
6. SAT radiodensity (HU)

The formula used to calculate the surface area (cm^2^) of a particular body composition for each slice was (size of the specific body composition × the pixel spacing). The pixel spacing was derived from the data included within each CT DICOM file.

The radiodensity of a specific body composition measure was determined by averaging the values of pixel representing that body composition in each slice.

### 2.3. AI Model

A two-dimension U-Net convolutional network that was trained and validated on 541 previously collected mid-L3 CT scans was used to segment muscle, VAT, and SAT (submitted for publication). The training dataset comprised 338 CT scans derived from CT scans of 116 CRC patients. Each patient's accessible CT scans (from six months prior to surgery or three months after surgery) were collected so that one or more scans were available for the same patient. For each patient, a trained human grader (author JoY) manually selected the mid-L3 CT slice based on the Alberta Protocol (https://tomovision.com/SarcopeniaHelp/index.html). Using a semiautomated software (Slice-O-Matic version 5.0, Tomovision, Quebec, Canada), all CT scans of the training dataset were manually segmented in accordance with the Alberta Protocol (https://tomovision.com/Sarcopenia_Help/index.html). This dataset was then randomly divided into a training (80% of scans, number of scans = 270) and a validation dataset (the remaining 20% of scans, number of scans = 68). The training dataset was used to develop the segmentation model, and the validation dataset was applied to assess the performance of the final fitted model. According to the results, the average dice coefficient in the validation dataset for all body composition segmentation was 0.98, with 0.98 for muscle, 0.98 for VAT, and 0.99 for SAT. The AI model was further tested on an additional CT dataset from another 203 patients, with 1 in 10 scans (number of scans = 21) selected at random for manual segmentation in order to perform cross-validation. The average dice coefficient for the AI model constructed in this test dataset was 0.98, with 0.97 for muscle, 0.98 for VAT, and 0.98 for SAT.

**Figure 1:**
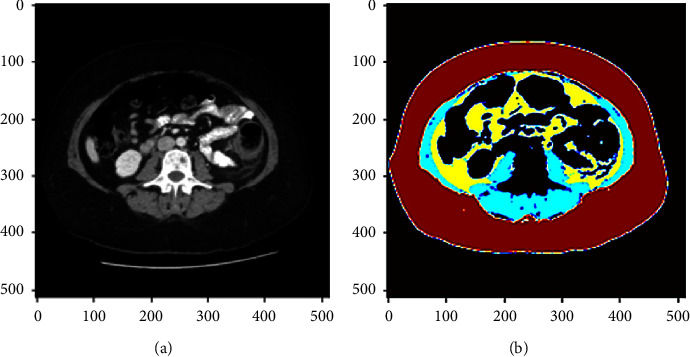
A sample case demonstrating the original CT scan (a) and the AI segmented CT slice (b). In the segmented CT slice, the red indicates the region of SAT, the yellow indicates the region of VAT, and the blue indicates the region of muscles.

Figure 1 shows an example of body composition segmentation, including an original CT scan and a segmented CT scan.

To assess the performance of our AI model in segmenting different L3 slices in the current dataset, all available scans at the L3 level (198 CT slices in total) from a randomly selected 21 patients were manually segmented (author JoY) using the semiautomated software (Slice-O-Matic version 5.0, Tomovision, Quebec, Canada), according to the Alberta Protocol (https://tomovision.com/Sarcopenia_Help/index.htm). The threshold settings for the segmentation tool were as follows: SM: −29 to 150 HU, VAT: −150 to −50 HU, and SAT: −190 to −30. These thresholds were predefined in the Alberta Protocol for SliceOmatic (https://tomovision.com/Sarcopenia_Help/index.htm).

The Sorensen–Dice coefficient (Dice coefficient) was used to determine the effectiveness of U-Net-based segmentation by comparing AI and manual reading on the 198 assessed scans. The average Dice coefficient achieved for all body composition segmentation on these scans was 0.97, with 0.97 for SM, 0.96 for VAT, and 0.97 for SAT, respectively, indicating that our AI produced a highly accurate representation of body composition segmentation for each of the different L3 slices.

### 2.4. Statistical Analysis

To compare body composition between mid-L3 and other L3 slices, the average percent difference was calculated. For a particular body composition measure of each patient, the average percent difference was computed using the formula: average (absolute value ((each L3 slice (excluding mid-L3) body composition–mid-L3 body composition)/mid-L3 body composition) × 100).

The Mann–Whitney test was performed to determine if there was a statistically significant difference between sexes (unpaired data) regarding continuous parameters. A *p* value threshold of 0.05 indicated a statistically significant result.

## 3. Results

The dataset for the current study consisted of 2203 CT scans obtained from 203 patients who had surgical treatment for CRC. The mean age of the cohort was 60.87 ± 12.42 years (97 M, 106 F). The median number of CT slices that represented the whole-L3 vertebra was 10 slices per patient (IQR: 9–11).

### 3.1. Single Mid-L3 Slice

Body composition measurements using the mid-L3 CT slice of all patients are shown in Table 1. Females had significantly less SM and VAT surface area than males (*p* < 0.001). Female patients exhibited significantly more SAT surface area and lower SAT density than male patients (*p* < 0.001).

### 3.2. Non-Mid-L3 versus Mid-L3 Slice

The average percent difference in SM, VAT, and SAT surface area and radiodensity between the mid-L3 slice and non-mid-L3 slices were calculated for each patient (Table 2, Supplementary Figure 1). Among these various body compositions, the VAT surface area had the greatest average percent difference (median = 6.28%, IQR = 3.94–10.79) between mid-L3 and non-mid-L3, followed by SAT surface area (median = 5.49%, IQR = 3.30–7.35), and SM surface area (median = 3.58%, IQR = 2.62–4.66).

We further examined the average percent difference in calculated measures of each body composition between the mid-L3 slice and the non-mid-L3 slices by sex (Table 2, Supplementary Figure 2). Female patients had a significantly larger average percent difference in VAT surface area (*p* = 0.02; median = 6.90%, IQR = 4.62–11.27) than males (median = 5.23%, IQR = 3.33–8.99). In contrast, male patients showed significantly larger percent differences in SAT surface area (*p* < 0.001, median = 6.60%, IQR = 4.64–8.31) (median = 4.33%, IQR = 2.38–6.22) and radiodensity (*p* = 0.007, median = 0.97%, IQR = 0.65–1.35) than females (median = 0.76%, IQR = 0.50–1.13).

## 4. Discussion

Body composition measurements, in particular SM surface area, have been associated with rectal cancer response to neoadjuvant therapy and corresponding survival outcomes [23, 24]. Furthermore, body composition has been suggested as a superior method of dosing chemotherapy for CRC, to decrease rates of dose-limiting toxicity [8, 25]. Currently, 2D body composition is still commonly measured as there is limited clinically validated software available for researchers and clinicians to use. As a result, the gold standard Alberta Protocol derived mid-L3 vertebral CT slice is routinely utilised for the measurement of body composition [10–12].

Two studies by Shen et al. [13, 14] published in 2004 have been frequently cited as justifications for the use of the L3 vertebra as the gold standard of obtaining body composition. The first study examined the relationship between cross-sectional VAT areas at various anatomic locations and VAT volume in 320 healthy subjects. Their findings indicated that the area between 5 and 10 cm above the L4-5 vertebrae level provided the most accurate estimate of VAT volume in men and women, respectively, when utilising only a single 2D CT slice. The latter study by Shen investigated the relationship between a single cross-sectional area at different anatomic locations and the total volume of muscle and adipose tissues in 328 healthy subjects. These results indicated that the area between 5 cm above the L4-5 level and 5 cm below the L4-5 level showed the highest correlation with muscle and adipose tissues volume, respectively. However, both studies relied on MRI scans, and neither study included CRC patients nor specifically stated the significance of L3 segments (although L3 is located 5/10 cm above L4-5). Another study by Schweitzer et al. [26] reported that a single MRI scan at the L3 level was the best representative site for assessing total volumes of SM, VAT, and SAT. Again, this study was conducted on only 142 healthy subjects and not CRC patients. Consequently, if considering using only a single representative CT slice for body composition, using a mid-L3 CT slice and correlating it to a patient's clinical outcome does not appear to have been adequately addressed and requires further investigation.

Our study demonstrated that body composition measurements obtained from a single-CT slice image at the mid-L3 vertebral level differ to those obtained from analysis of multiple slices that constitute the entire L3 vertebra. The surface area of body composition components displayed a large degree of variability across L3. For example, VAT and SAT surface area readings had a median of 5.49% and 6.28% in average percent difference, respectively, between non-mid-L3 slices and the mid-L3 vertebral slice.

It was of particular interest that we identified significant variation in body composition parameters in the mid-L3 slice and the non-mid-L3 slices between the two sexes. Our study also demonstrated that between the mid-L3 slice and non-mid-L3 slices, VAT variance was greater in females, whereas the opposite was true for SAT variance.

From our results, it can be surmised that the use of only a single 2D CT scan at the mid-L3 level presents a limited view of body composition and that the advent of AI now offers researchers an enhanced and more accurate means of obtaining a broader based measure of 3D body composition measures which will aid in our understanding of the role that body composition plays in clinical outcomes.

In this study, we have presented results from our validated AI model to automatically segment body composition measures for SM, SAT, and VAT from multiple CT slices across the whole-L3 vertebra in CRC patients. Manual cross-check validation with experienced researchers demonstrated that the AI model provides excellent body composition segmentation on all CT slices at this L3 level (Dice similarity of 0.97).

Despite these promising results, there were several limitations to our study. The study was conducted at a single centre, with data that were collected retrospectively. Furthermore, these findings on body composition measures need to be further elaborated on their clinical impact on CRC outcomes. In addition, while our results are highly promising, we should note that our results have not been evaluated on an external dataset (i.e., other hospital institutions or in other countries). Our future work will recruit additional internal and external patient datasets to test the validity of our results and strengthen our findings with data from various institutions and patient cohorts in order to verify its robustness. A future prospective study in a clinical context is essential to conduct more rigorous testing of our AI models, specifically to evaluate their generalizability and robustness.

## 5. Conclusion

We found that the use of multiple CT slices from various locations on L3 identified significant variations in estimates of body composition compared to when only using a single slice from the mid-L3 vertebral level. This heterogeneity in body composition across L3 was significantly linked to sex differences. The use of AI to derive 3D body composition offers an enhanced means of obtaining a more accurate measure of body composition as a predictive tool for determining outcomes related to colorectal cancer.

## Figures and Tables

**Table 1:** Characteristics of body composition for all patients and by sex in CRC patients using only the mid-L3 slice.

	All (*n* = 203)	*Sex split*		
		Female (*n* = 106 patients)	Male (*n* = 97 patients)	*p* value (male vs female)
	Median (interquartile range)			
SM surface area (cm^2^)	125.03 (101.69–149.00)	103.92 (96.31–119.66)	148.14 (133.44–164.21)	**<0.001**
Muscle radiodensity (HU)	37.82 (31.63–43.44)	37.07 (30.60–43.21)	38.78 (33.13–44.11)	0.14
VAT surface area (cm^2^)	138.12 (80.76–225.92)	107.68 (69.88–174.59)	191.34 (99.64–282.73)	**<0.001**
VAT radiodensity (HU)	−90.89 (−95.70–−84.34)	−90.52 (−95.27–−84.20)	−91.36 (−97.00–−85.10)	0.51
SAT surface area (cm^2^)	199.30 (132.30–292.60)	262.68 (171.61–346.77)	148.0 (108.5–201.7)	**<0.001**
SAT radiodensity (HU)	−102.74 (−106.15–−95.69)	−103.92 (−107.85–−100.17)	−99.37 (−104.29–−92.26)	**<0.001**

**Table 2:** Average percent difference (%) in muscle, VAT, SAT area, and radiodensity between the mid-L3 slice and non-mid-L3 slices in all patients and by sex. For each patient, the average percent difference was calculated by averaging (absolute value ((each L3 slice (excluding mid-L3) body composition–mid-L3 body composition)/mid-L3 body composition) × 100).

	All (*n* = 203)	*Sex split*		
		Female (*n* = 106 patients)	Male (*n* = 97 patients)	*p* value (male vs female)
	Average percent difference (%) median (interquartile range)			
SM surface area	3.58% (2.62–4.66)	3.64% (2.62–4.67)	3.54% (2.57–4.63)	0.96
Muscle radiodensity	2.85% (2.00–4.00)	3.02% (2.03–4.26)	2.74% (1.89–3.71)	0.09
VAT surface area	6.28% (3.94-10.79)	6.90% (4.62-11.27)	5.23% (3.33–8.99)	**0.02**
VAT radiodensity	1.28% (0.86–2.04)	1.32% (0.85–2.05)	1.23% (0.90–2.00)	0.85
SAT surface area	5.49% (3.30–7.35)	4.33% (2.38–6.22)	6.6% (4.64–8.31)	**<0.001**
SAT radiodensity	0.85% (0.57–1.27)	0.76% (0.50–1.13)	0.97% (0.65–1.35)	**0.007**

## Data Availability

The datasets generated during and/or analysed during the current study are available from the corresponding author on reasonable request.

## Supplementary Materials

Supplementary Materials Supplementary Figure 1 Average percent difference in muscle, VAT, and SAT surface area and radiodensity between the mid-L3 slice and non-mid-L3 slices for all patients. Supplementary Figure 2 Average percent difference in muscle, VAT, and SAT surface area and radiodensity between the mid-L3 slice and non-mid-L3 slices for females (A) and males (B).

## References

[1] Bedrikovetski S., Seow W., Kroon H. M., Traeger L., Moore J. W., Sammour T. (2022). Artificial intelligence for body composition and sarcopenia evaluation on computed tomography: a systematic review and meta-analysis. *European Journal of Radiology*.

[2] Bredella M. A. (2017). Sex differences in body composition. *Advances in Experimental Medicine and Biology*.

[3] Malietzis G., Currie A. C., Athanasiou T. (2016). Influence of body composition profile on outcomes following colorectal cancer surgery. *British Journal of Surgery*.

[4] Almasaudi A. S., Dolan R. D., McSorley S. T., Horgan P. G., Edwards C., McMillan D. C. (2019). Relationship between computed tomography-derived body composition, sex, and post-operative complications in patients with colorectal cancer. *European Journal of Clinical Nutrition*.

[5] Drami I., Pring E. T., Gould L. (2021). Body composition and dose-limiting toxicity in colorectal cancer chemotherapy treatment; a systematic review of the literature. Could muscle mass be the new body surface area in chemotherapy dosing?. *Clinical Oncology*.

[6] Hopkins J. J., Reif R. L., Bigam D. L., Baracos V. E., Eurich D. T., Sawyer M. B. (2019). The impact of muscle and adipose tissue on long-term survival in patients with stage I to III colorectal cancer. *Diseases of the Colon and Rectum*.

[7] Xiao J., Mazurak V. C., Olobatuyi T. A., Caan B. J., Prado C. M. (2018). Visceral adiposity and cancer survival: a review of imaging studies. *European Journal of Cancer Care*.

[8] Cao K., Yeung J., Arafat Y. (2023). Can AI-based body composition assessment outperform body surface area in predicting dose-limiting toxicities for colonic cancer patients on chemotherapy?. *Journal of Cancer Research and Clinical Oncology*.

[9] Yip C., Dinkel C., Mahajan A., Siddique M., Cook G. J., Goh V. (2015). Imaging body composition in cancer patients: visceral obesity, sarcopenia and sarcopenic obesity may impact on clinical outcome. *Insights Imaging*.

[10] Arayne A. A., Gartrell R., Qiao J., Baird P. N., Yeung J. M. (2023). Comparison of CT derived body composition at the thoracic T4 and T12 with lumbar L3 vertebral levels and their utility in patients with rectal cancer. *BMC Cancer*.

[11] Brown J. C., Heymsfield S. B., Caan B. J. (2022). Scaling of computed tomography body composition to height: relevance of height-normalized indices in patients with colorectal cancer. *J Cachexia Sarcopenia Muscle*.

[12] Kotti A., Holmqvist A., Woisetschläger M., Sun X.-F. (2022). Computed tomography-measured body composition and survival in rectal cancer patients: a Swedish cohort study. *Cancer and Metabolism*.

[13] Shen W., Punyanitya M., Wang Z. (2004). Visceral adipose tissue: relations between single-slice areas and total volume. *The American Journal of Clinical Nutrition*.

[14] Shen W., Punyanitya M., Wang Z. (1985). Total body skeletal muscle and adipose tissue volumes: estimation from a single abdominal cross-sectional image. *Journal of Applied Physiology*.

[15] Thompson W., Li H., Bolen A. (2020). *Artificial Intelligence, Machine Learning, Deep Learning and beyond*.

[16] Lee H., Troschel F. M., Tajmir S. (2017). Pixel-level deep segmentation: artificial intelligence quantifies muscle on computed tomography for body morphometric analysis. *Journal of Digital Imaging*.

[17] Weston A. D., Korfiatis P., Kline T. L. (2019). Automated abdominal segmentation of CT scans for body composition analysis using deep learning. *Radiology*.

[18] Bridge C. P., Rosenthal M., Wright B. (2018). Fully-automated analysis of body composition from CT in cancer patients using convolutional neural networks. *OR 2.0 Context-Aware Operating Theaters, Computer Assisted Robotic Endoscopy, Clinical Image-Based Procedures, and Skin Image Analysis*.

[19] Burns J. E., Yao J., Chalhoub D., Chen J. J., Summers R. M. (2020). A machine learning algorithm to estimate sarcopenia on abdominal CT. *Academic Radiology*.

[20] Park H. J., Shin Y., Park J. (2020). Development and validation of a deep learning system for segmentation of abdominal muscle and fat on computed tomography. *Korean Journal of Radiology*.

[21] Paris M. T., Tandon P., Heyland D. K. (2020). Automated body composition analysis of clinically acquired computed tomography scans using neural networks. *Clinical Nutrition*.

[22] Khan S., Warkhedkar R., Shyam A. (2014). Analysis of Hounsfield unit of human bones for strength evaluation. *Procedia Materials Science*.

[23] Liu Z., Lu S., Wang Y. (2022). Impact of body composition during neoadjuvant chemoradiotherapy on complications, survival and tumor response in patients with locally advanced rectal cancer. *Frontiers in Nutrition*.

[24] Wei M. Y., Arafat Y., Lee M. (2023). Emerging trends in the prediction of pathological tumour response in rectal cancer following neoadjuvant therapy. *ANZ Journal of Surgery*.

[25] Arafat Y., Loft M., Cao K. (2022). Current colorectal cancer chemotherapy dosing limitations and novel assessments to personalize treatments. *ANZ Journal of Surgery*.

[26] Schweitzer L., Geisler C., Pourhassan M. (2015). What is the best reference site for a single MRI slice to assess whole-body skeletal muscle and adipose tissue volumes in healthy adults?. *The American Journal of Clinical Nutrition*.

