# Supplementary figures and images for: Identification of Differences in Body Composition Measures Using 3D-Derived Artificial Intelligence from Multiple CT Scans across the L3 Vertebra Compared to a Single Mid-Point L3 CT Scan
Source: Radiol Res Pract. 2023 Oct 17;2023:1047314. doi: 10.1155/2023/1047314 (PMC10597731; doi:10.1155/2023/1047314)

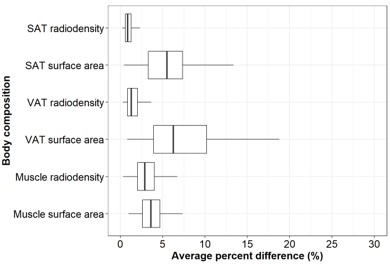

Supplement: Supplementary Materials — Supplementary Figure 1 Average percent difference in muscle, VAT, and SAT surface area and radiodensity between the mid-L3 slice and non-mid-L3 slices for all patients. Supplementary Figure 2 Average percent difference in muscle, VAT, and SAT surface area and radiodensity between the mid-L3 slice and non-mid-L3 slices for females (A) and males (B). [file 1047314.f1.zip › Supplementary Figure 1.jpg]

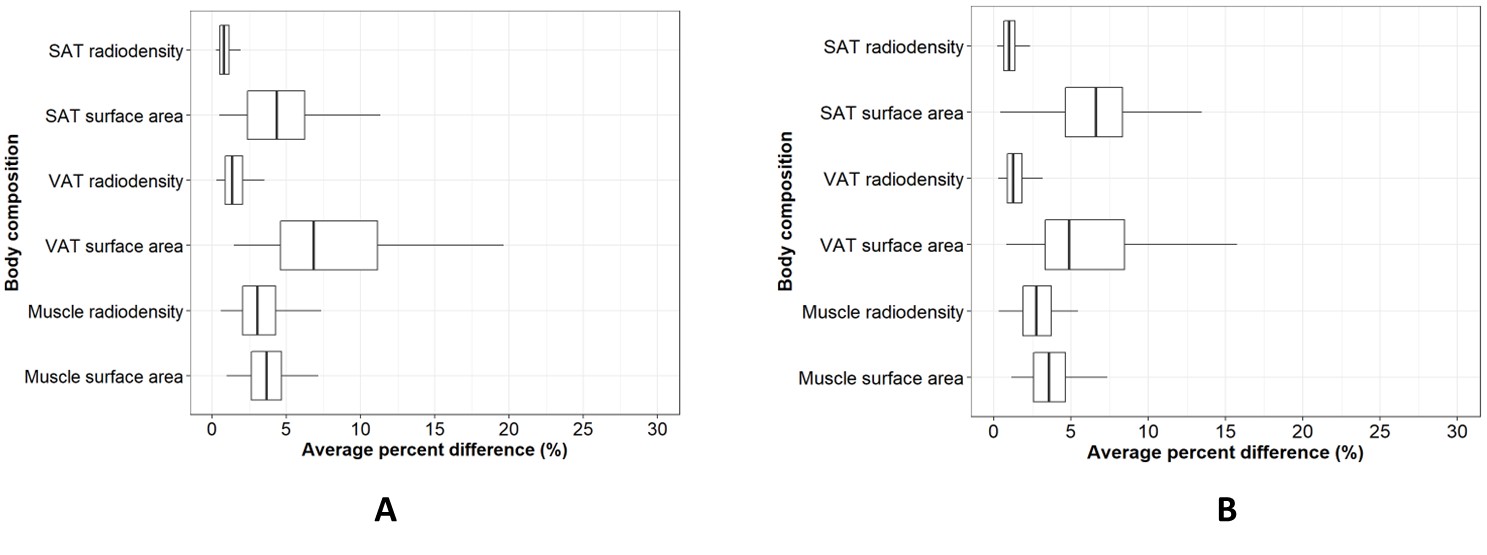

Supplement: Supplementary Materials — Supplementary Figure 1 Average percent difference in muscle, VAT, and SAT surface area and radiodensity between the mid-L3 slice and non-mid-L3 slices for all patients. Supplementary Figure 2 Average percent difference in muscle, VAT, and SAT surface area and radiodensity between the mid-L3 slice and non-mid-L3 slices for females (A) and males (B). [file 1047314.f1.zip › Supplementary Figure 2.jpg]
